# Novel Optical Criteria and Mechanisms of Critical Decline in Liver Regenerative Potential

**DOI:** 10.3390/cells13232015

**Published:** 2024-12-05

**Authors:** Svetlana Rodimova, Vera Kozlova, Nikolai Bobrov, Dmitry Kozlov, Artem Mozherov, Vadim Elagin, Ilya Shchechkin, Dmitry Kuzmin, Alena Gavrina, Vladimir Zagainov, Elena Zagaynova, Daria Kuznetsova

**Affiliations:** 1 Institute of Experimental Oncology and Biomedical Technologies, Privolzhsky Research Medical University, 10/1 Minin and Pozharsky Sq., 603000 Nizhny Novgorod, Russiamail-kozlov2015@yandex.ru (D.K.); ezagaynova@gmail.com (E.Z.); daria.s.kuznetsova@gmail.com (D.K.); 2 Department of Molecular Biology and Immunology, Lobachevsky Nizhny Novgorod National Research State University, Gagarina 23, 603022 Nizhny Novgorod, Russia; 3 The Volga District Medical Centre of Federal Medical and Biological Agency, 14 Ilinskaya Str., 603000 Nizhny Novgorod, Russia; 4 Laboratory of Omics and Regenerative Technologies, Institute for Regenerative Medicine, Sechenov First Moscow State Medical University (Sechenov University), 8–2 Trubetskaya Str., 119991 Moscow, Russia; 5 Nizhny Novgorod Regional Clinical Oncologic Dispensary, Delovaya Str., 11/1, 603126 Nizhny Novgorod, Russia; 6 Lopukhin Federal Research and Clinical Center of Physical-Chemical Medicine of Federal Medical Biological Agency, 1a Malaya Pirogovskaya Str., 119435 Moscow, Russia

**Keywords:** liver regeneration, acute liver injury, multiphoton microscopy, FLIM, SHG

## Abstract

The most effective method of treating tumors localized in the liver remains resection. However, in the presence of concomitant pathology, the regenerative potential of the liver is significantly reduced. To date, there is insufficient fundamental data on the mechanisms responsible for the disruption of liver regeneration, and there is no effective method for assessing its regenerative potential. The most suitable model for these purposes is acute liver injury (ALI). Modern non-contrast methods of multiphoton microscopy with second harmonic generation and fluorescence lifetime imaging microscopy (FLIM) modes enable intravital evaluation of the metabolic status of the hepatocytes; therefore, this expands the possibilities for studying the processes occurring in cells during regeneration in the context of any pathologies.

## 1. Introduction

A healthy liver has an outstanding ability to recover from chemical or mechanical damage. However, with accompanying acute or chronic liver disease, there is a significant decrease in the regenerative potential of the liver. Assessing the liver’s ability to recover is an important task when planning the extent of resection in the clinic [1]. In this regard, there is a continuing search for new criteria to identify reduced liver regenerative potential in pathology. A significant contribution to liver pathogenesis and to decrease in its regenerative potential is the result of changes in the energy metabolism of hepatocytes. Hepatocytes make up 70–80% of the liver parenchyma and are central to the metabolism of fats, proteins and carbohydrates.

To search for new criteria indicating liver condition, in this work, we propose the use of acetaminophen (APAP)-induced ALI. ALI is a complex clinical syndrome characterized by a transient loss of liver function with a high risk of death, reaching 7.4–11.9% [2,3,4,5,6] due to its rapid development, which therefore requires urgent hospitalization [7]. The key characteristic of this model is the critical impairment of the liver’s ability to regenerate, thus enabling the collection of new fundamental data and the identification of new optical criteria for determining the violated liver’s reduced regenerative potential.

In contemporary biomedical investigations, fluorescence bioimaging techniques, including multiphoton microscopy with SHG and FLIM, are being extensively used. These techniques are label-free, minimally invasive, and facilitate intravital visualization of tissue structure at a cellular level. Furthermore, they are based on data obtained on the fluorescence lifetimes of various forms of intracellular co-factors, in particular, nicotinamide adenine dinucleotide (NADH) and its phosphorylated form NADPH [8,9,10,11]. In contrast, NADPH plays a role in numerous biosynthetic pathways, such as the pentose phosphate pathway and lipogenesis, and also contributes to antioxidant defense, particularly in the glutathione cycle [12].

Thus, in this work, we studied the metabolic state of hepatocytes and obtained new optical criteria for identifying a critical decrease in the regenerative potential of the liver in the ALI model using these modern methods (SHG and FLIM) of multiphoton microscopy. The results obtained will be relevant from both fundamental and applied points of view, due to the possibility of translation of the obtained optical features for use in clinical situations.

## 2. Materials and Methods

### 2.1. Animal Model

A series of experiments was conducted, involving 68 male Wistar rats weighing between 300 and 400 g. ALI was induced through the intraperitoneal administration of APAP solution at a dosage of 1 g/kg of body weight. Liver regeneration was prompted by performing either a 30% partial hepatectomy (30% PH) or a 70% partial hepatectomy (70% PH) after 24 h from the administration of APAP. Following PH, each rat was placed in a sanitized cage and maintained under standard conditions in a specific pathogen-free (SPF) vivarium. To evaluate the liver condition with ALI before initiating liver regeneration, we analyzed ex vivo liver samples collected during the resection (0 day). The remaining samples were examined on the 3rd and 7th days following the PH.

Ex vivo samples were obtained immediately after PH (0 day) or after isolation of the whole organ (on the 3rd and 7th day of regeneration). Initially, the liver was washed with saline. Immediately after obtaining the ex vivo sample, we performed studies using multiphoton microscopy, SHG, and FLIM. The time required for obtaining images did not exceed 30 min from the moment the ex vivo sample was collected.

**Figure 1:**
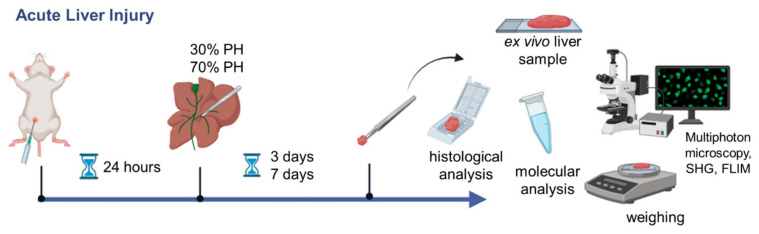
Road map of the steps in the experiment on liver regeneration with induced ALI.

The scheme of the experiment is presented in Figure 1.

Four biological groups were distinguished. 1. rats with normal liver—30% PH model (0 day n = 24, 3rd day n = 12, 7th day n = 12); 2. rats with normal liver—70% PH model (0 day n = 24, 3rd day n = 12, 7th day n = 12); 3. rats with ALI—30% PH model (0 day n = 10, 3rd day n = 5, 7th day n = 5); 4. rats with ALI—70% PH model (0 day n = 10, 3rd day n = 5, 7th day n = 5).

The animals were kept both prior to and following the surgery in accordance with the regulations established by the European Convention for the Protection of Vertebrate Animals, as well as the National Institutes of Health Guide for the Care and Use of Laboratory Animals (NIH Publications No. 8023, revised 1978), and in alignment with the ARRIVE guidelines. All animal procedures received approval from the local ethics committee at Privolzhsky Research Medical University (protocol No. 2; date: 17 February 2023).

### 2.2. Morphological Analysis

For the histological examinations, the liver was preserved in a 10% buffered formalin solution, processed through isopropyl alcohol, and embedded in paraffin. Deparaffinized sections measuring 7 μm were stained with hematoxylin/eosin following the conventional protocol [13]. Sections were imaged using an all-in-one type EVOS M7000 Imaging System (Thermo Fisher Scientific Inc., Waltham, MA, USA).

To assess the effectiveness of liver recovery, we calculated the percentage of liver recovery at different stages of the regeneration. For this, before the start of regeneration (the starting point) and at different stages of the recovery process, the ratio of liver weight to animal body weight was measured, and then the percentage of restored liver weight was calculated. To calculate the initial liver weight we used the following formulae: *weight of the resected liver (g)/0.3* for the 30% PH model, and *weight of the resected liver (g)/0.7* for the 70% PH model.

The formula used to calculate the percentage of liver weight recovery was the following: *liver remnant weight (g)/body weight after PH (g)/initial liver weight (g)/body weight before PH (g)*—this value was taken as the absolute percentage of liver weight recovery (“absolute weight”). To assess the percentage of liver recovery in induced ALI in relation to the corresponding day of normal regeneration, we calculated the relative percentage of liver mass recovery (“relative weight”). For this, we calculated the ratio of the absolute percentage of liver weight recovery in ALI (absolute weight_ALI_) to the absolute percentage of liver weight recovery of normal liver (absolute weight_norm_) for the corresponding time point of normal regeneration: *absolute weight_ALI_ (g)/absolute weight_norm_ (g)* × *100%*.

Morphometric analysis was conducted on histological sections stained with hematoxylin and eosin, evaluating the following indicators: the quantity of tetraploid hepatocytes (cells characterized by brightly colored, enlarged nuclei), the count of binucleate cells, and the number of mitotic cells. The average values for all these parameters were determined in relation to 100 normal cells [14].

### 2.3. Biochemical Blood Tests

Blood samples were obtained from the tail vein of each rat. The blood was then centrifuged at 3000 rpm for 15 min to obtain the serum. The following parameters were evaluated: the levels of aspartate aminotransferase (AST), alanine aminotransferase (ALT), alkaline phosphatase (ALP), total protein, creatinine, and urea. The analysis was carried out using an automated biochemical analyzer (Mindray BS-120, Shenzhen, China) and standard reagents (Vital, Saint Petersburg, Russia), in accordance with the manufacturer’s protocol.

### 2.4. FLIM Analysis

The examination of all samples was conducted using an LSM 880 (Carl Zeiss, Berlin, Germany) equipped with a Ti:Sapphire femtosecond laser (repetition rate: 80 MHz, pulse duration less than 100 fs) and a time-correlated single photon counting (TCSPC) system (Simple-Tau 152, Becker Hickl GmbH, Berlin, Germany). The average laser power utilized was approximately 10 mW. A C-Plan-Apochromat 40×/1.3 oil immersion objective was employed to capture the fluorescence signals. For each sample, fluorescence intensity images of NAD(P)H and FLIM data were obtained from 10 fields of view. NAD(P)H fluorescence was excited at λex = 750 nm, with emission detected in the range of λem = 450–490 nm. The SHG signal was generated at 800 nm, with detection occurring in the 371–421 nm range. The FLIM analysis was carried out using SPCImage 8.3 software (Becker Hickl GmbH, Berlin, Germany), using a tri-exponential decay model. To ensure a minimum of 10,000 counts per pixel, the binning parameter was adjusted to 2. The goodness of fit for the model was evaluated by the χ2 value (which should be around 1). The following parameters were analyzed in 20–30 regions of the cell cytoplasm for each field of view: tm (ps), the amplitude-weighted mean fluorescence lifetimes; the relative contributions of free NADH, a1 (%), bound NADH, a2 (%), and NADPH, a3 (%).

### 2.5. Real-Time PCR

Isolation of total RNA from the samples was performed according to the protocol for the HiPure Total RNA Kit (#R401103, Magen, Guangzhou, China). Before the reverse transcription reaction, the samples were treated with DNAase TURBO DNA-free™ Kit (#AM1907, Invitrogen, Waltham, MA, USA). Real-time PCR was performed on a Bio-Rad CFX96 machine (Bio-Rad, Hercules, CA, USA) using a reaction mixture based on SYBR Green (#7567 Invitrogen, Waltham, MA, USA). The PCR reaction mixture contained 1× GeneAmp PCR Buffer I (#8080129, Applied Biosystems, Waltham, MA, USA), 250 µM of each dNTP, 0.5 nM of each primer, and 1 U of Taq M polymerase (#751-100, Intifica, Saint Petersburg, Russia). The total concentration of Mg2+ in the reaction was 3 mM, the reaction volume being 20 µL. The temperature profile of all cycles was as follows: (1) 95 °C for 10 min (enzyme activation step), (2) 40 cycles of 95 °C for 15 s, 60 °C for 30 s, and 72 °C for 30 s, (3) hybridization 1 min at 95 °C and 1 min at 40 °C, and (4) melt curve analysis with measurements between 60 °C and 95 °C. The reaction efficiency was calculated using a standard curve. The following reference genes were used for expression normalization: HPRT and GSL (GeNorm V < 0.15). Quantitative RT-PCR analysis was performed using CFX Maestro 2.3 software.

### 2.6. Statistics

To assess the statistical significance of changes in the regenerative capacity of liver with ALI, the nonparametric Mann–Whitney U test was employed. The statistical analysis was conducted using R programming software version 2024.09.01+394. Comparative analyses were carried out between the recovered liver weights with induced ALI on the 3rd and 7th days post 30% or 70% PH and the corresponding time points of normal liver regeneration; *p*-value ≤ 0.05.

At each time point, we obtained 8–10 fluorescence and FLIM images of NAD(P)H. For each image, we quantitatively evaluated the NAD(P)H fluorescence intensity in the cytoplasm of 30 hepatocytes (in regions with high NAD(P)H fluorescence intensity) and in 10–30 hepatocytes (in areas with low NAD(P)H fluorescence intensity), excluding the nucleus. For each FLIM image, we analyzed the FLIM parameters in the cytoplasm of 30 hepatocytes. Statistical analysis was performed using R software version 2024.09.01+394. Shapiro–Wilkes tests and F-tests indicated a normal distribution of the FLIM data. For pairwise comparisons, we utilized the t-test method with Bonferroni correction; *p*-value ≤ 0.05.

The all graphs were executed in Python 3.11.8 (Python Software Foundation, Wilmington, DE, USA) using the Visual Studio Code 1.95 (Microsoft, OneMicrosoft Way Redmond, Washington, DC, USA) development environment.

## 3. Results

### 3.1. Multiphoton Microscopy

**Figure 2:**
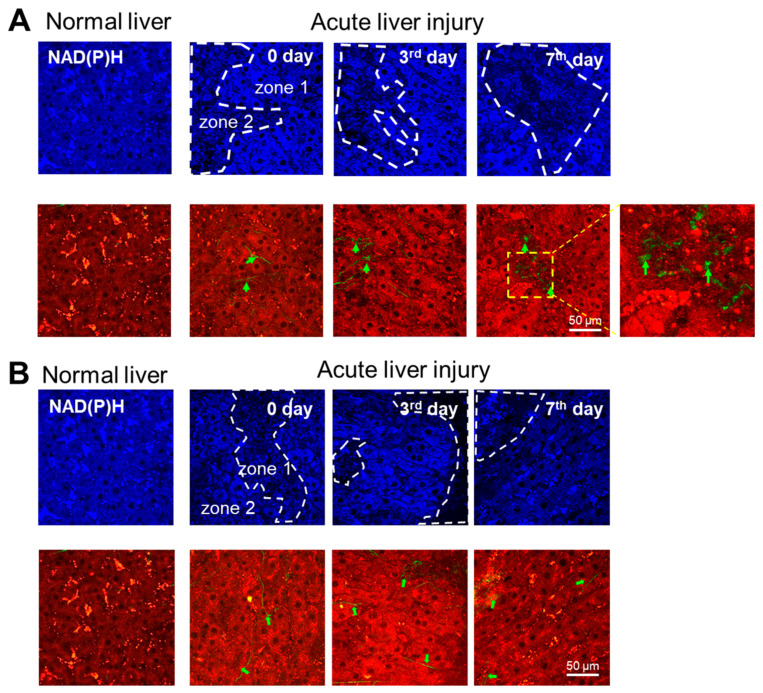
Analysis of the structural state of liver tissue in normal state and with ALI during induced regeneration after (**A**) 30% PH and (**B**) 70% PH. Fluorescence intensity images of NAD(P)H fluorescence and the second harmonic generation of collagen (green) in liver tissue. NAD(P)H fluorescence: excitation at 750 nm, detection range 455–500 nm; cell autofluorescence (red): excitation at 800 nm, detection range 433–660 nm; SHG (green): excitation at 800 nm, detection range 371–421 nm. Green arrows indicate collagen. The dotted line indicates a zone with reduced NAD(P)H fluorescence intensity. Scale bar: 50 μm; ×400.

Using multiphoton microscopy in two-photon mode, we revealed a uniform distribution of NAD(P)H fluorescence intensity for both the 30% and 70% PHs during normal liver regeneration. SHG microscopy showed an absence of a second harmonic signal from collagen. The results are presented in Figure 2A,B.

With induced ALI for the 30% and 70% PH models in two-photon mode, we revealed zones with reduced NAD(P)H fluorescence intensities, which were associated with damaged hepatocytes and foci of fibrosis. The damaged hepatocytes were characterized by high lipid infiltration with bright inclusions of vitamin A [9]. The SHG microscopy revealed few foci of fibrosis and only mild accumulation of collagen fibers in the liver tissue. The results are presented in Figure 2A,B.

Interestingly, histological analysis revealed diffuse changes in the liver tissue, in particular, balloon dystrophy and vacuolization of the hepatocytes being distributed evenly throughout the liver tissue. However, using multiphoton microscopy, we could clearly distinguish between zones of hepatocytes with preserved metabolic activity (zone 1) and those with damaged hepatocytes (zone 2). This confirms that histological analysis does not provide information about intravital processes.

With induced ALI, during regeneration after 30% PH, the ratio of the intensity of the NAD(P)H fluorescence in both zone 1 and zone 2 varied from 3.20 ± 1.76 c.u. up to 4.18 ± 1.28 c.u., while after 70% PH, it varied from 3.36 ± 0.85 c.u. up to 4.66 ± 2.00 c.u.

### 3.2. FLIM Microscopy

**Figure 3:**
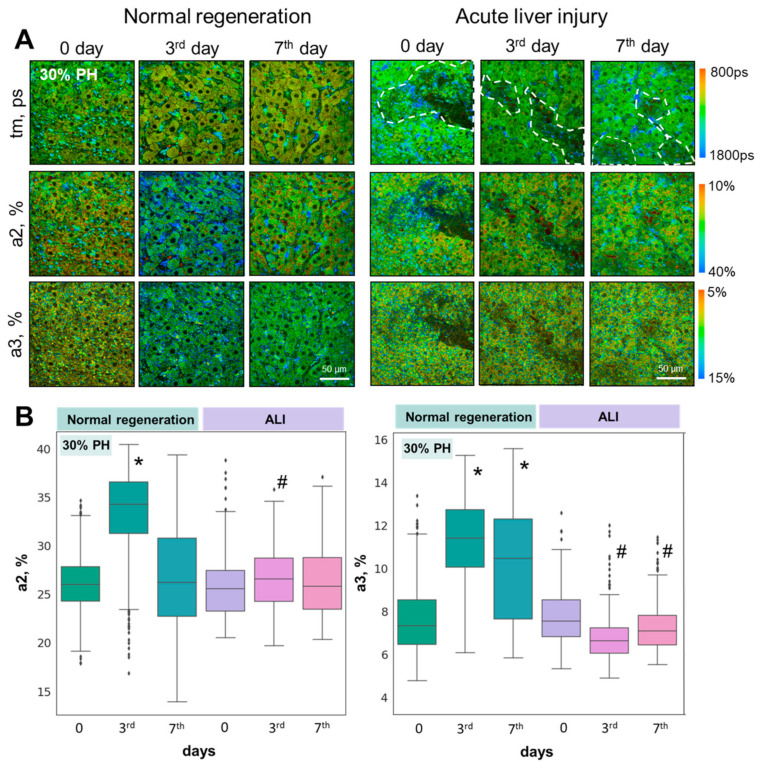
FLIM analysis of liver tissue with ALI during induced regeneration after 30% PH; (**A**) pseudo-coded FLIM images at different stages of liver regeneration in normal state and with ALI. (**B**) Boxplots reflecting the distribution of values of the fluorescence lifetime contributions of the bound forms of NADH and NADPH. Scale bar: 50 μm; ×400. *—statistically significant difference compared to normal liver (0 day). #—statistically significant differences compared to the corresponding time point for normal regeneration; *p*-value ≤ 0.05.

**Figure 4:**
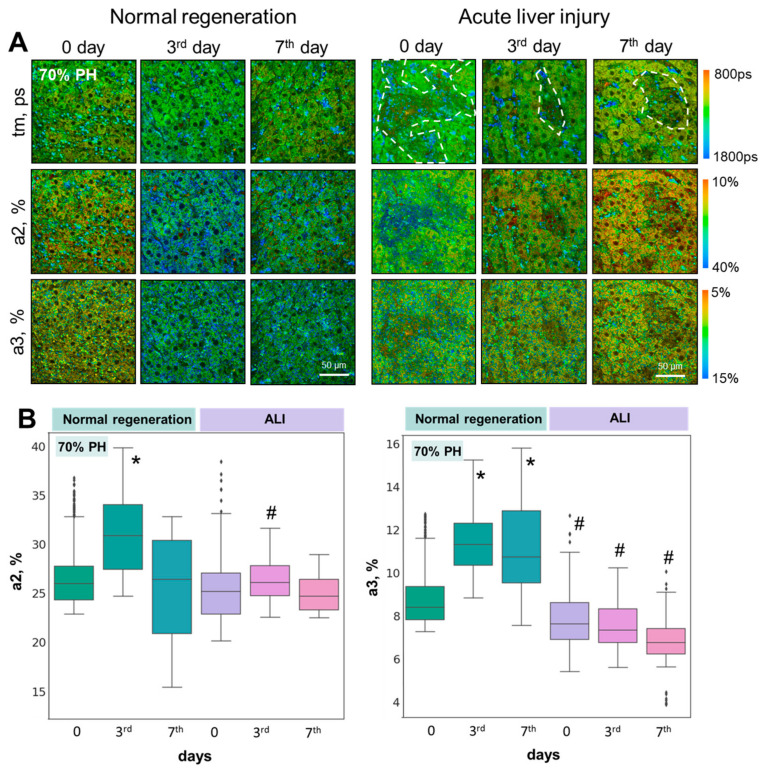
FLIM analysis of liver tissue with ALI during induced regeneration after 70% PH; (**A**) pseudo-coded FLIM images at different stages of liver regeneration in normal state and with ALI. (**B**) Boxplots reflecting the distribution of values of the fluorescence lifetime contributions of the bound forms of NADH and NADPH. Scale bar: 50 μm; ×400. *—statistically significant difference compared to normal liver (0 day). #—statistically significant differences compared to the corresponding time point for normal regeneration; *p*-value ≤ 0.05.

Using FLIM, we analyzed the contributions of the fluorescence lifetimes of the free and bound forms of NADH (a1% and a2%, respectively), and NADPH (a3%) (Figure 3A,B). The graphs show the values of the a2 and a3 percentages, as the most indicative parameters for assessing the metabolic state of hepatocytes under various conditions (Figure 3 and Figure 4).

It was shown that for both the 30% and 70% PH models with induced ALI, there was no sharp jump in the values of a2 and a3 on the 3rd day of regeneration (Figure 4A,B), as seen with normal regeneration. More specifically, it was shown that on the 3rd day of regeneration in the ALI model after 30% PH and 70% PH, the a2 values decreased by 13.5% and by 10.8%, respectively, relative to the 3rd day of normal liver regeneration.

We have previously shown that an increased intensity of OXPHOS and biosynthetic processes in hepatocytes on the 3rd day of regeneration is an optical criterion for the effectiveness of liver restoration [15].

Thus, the absence of a significant increase in a2 and a3 on the 3rd day of regeneration is a significant criterion indicating a greater impairment of the regenerative capacity of the liver with ALI. For the 70% PH model, we obtained even lower values of a2 and a3 in comparison with normal regeneration than those obtained for 30% PH. Such a result is consistent with the data previously obtained in a study on the regenerative potential of the liver with induced fibrosis and steatosis [16], as well as in cases with concomitant diabetes [17]. These pathologies were also characterized by a significant decrease in the regenerative potential of the liver.

### 3.3. Liver Weight Restoration Assessment

**Figure 5:**
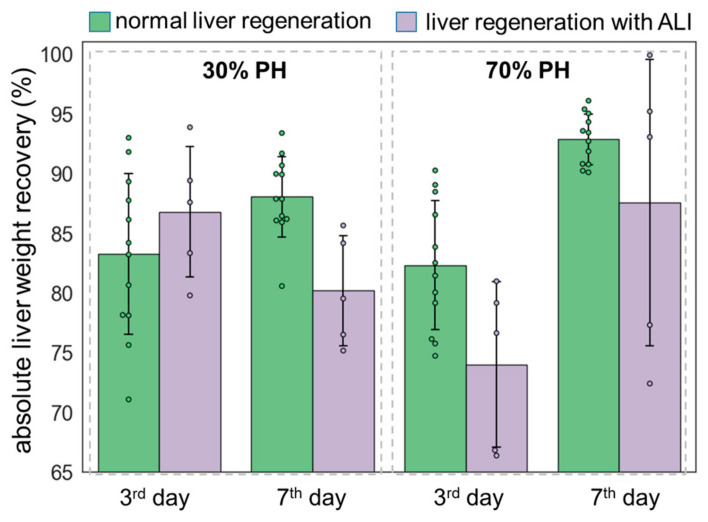
Analysis of liver weight recovery during regeneration in normal state and with induced ALI using 30% PH and 70% PH models. Each dot represents a measurement of the weight of one animal. Data are presented as mean ± standard deviation.

Liver recovery was assessed based on the percentage of liver weight recovery. The results of the analysis are presented in Figure 5 and in Table S1.

The percentage of liver weight recovery with induced ALI is almost equal to the corresponding percentage of liver recovery during normal regeneration. Despite the high percentage of liver weight recovery with concomitant ALI, the quality of the tissue differs from that of a normal liver. That is, the weight of the liver is restored not only due to the division of hepatocytes but also due to edema, as well as the accumulation of lipid droplets and collagen fibers.

### 3.4. Morphological Analysis and Biochemical Blood Tests

**Figure 6:**
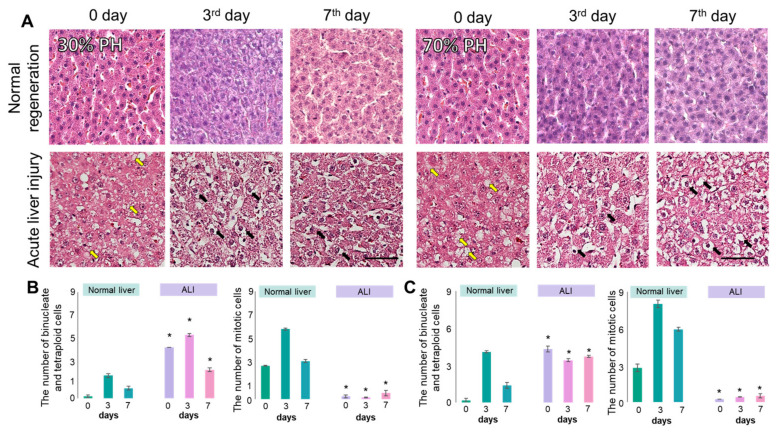
Histological (**A**) and morphometric analysis (**B**,**C**) of liver tissue with ALI during induced regeneration after 30% PH and 70% PH, hematoxylin and eosin; black arrows indicate balloon dystrophy of hepatocytes; yellow arrows indicate vacuolated hepatocytes. *—statistically significant differences compared to the corresponding time point for normal regeneration; *p*-value ≤ 0.05. Scale bar: 50 μm; ×400.

Histological analysis of the normal liver during regeneration showed an absence of pathological changes, except for an accumulation of lipid droplets in the hepatocytes, this being most pronounced at 70% PH (Figure 6A), which is characteristic of normal liver regeneration [9,18].

Before the induction of regeneration with concomitant ALI, we observed small- and medium-droplet vacuolar dystrophy. The nuclei were wrinkled, with weakly expressed perinuclear edema. The cell membranes of the hepatocytes were preserved, as was the tissue architecture. Such dystrophic changes are characteristic of ALI (Figure 6A) [19,20].

On the 3rd day after either 30% or 70% PH with ALI, we noted the hypertrophy of the hepatocytes, with a violation of the nuclear–cytoplasmic ratio, towards an increase in the volume of cell cytoplasm. The cell membranes of some cells were damaged, resulting in pericellular edema. The cytoplasm of the hepatocytes was also granular and vacuolated. On the 7th day for both models, focal colliquative and small focal necrosis could be observed. Within the circumference of necrosis, there was significant expansion of both the lumens of the sinusoids and of the Disse spaces, with a violation of the tissue architecture. The cytoplasm of the cells had signs of granular dystrophy, such as increased nuclear–cytoplasmic ratios and edema, with many acidophilic protein granules in the cytoplasm.

Morphometric analysis revealed a significant decrease in the number of mitotic cells during regeneration after 30% (Figure 6B) and 70% PH (Figure 6C) with induced ALI, in comparison with the regeneration of normal liver (Figure 6B,C). At the same time, the number of tetraploid and binucleate cells was significantly higher compared to the normal liver (Figure 6B,C), indicating a delay in the cell cycle, which is characteristic of liver pathology.

**Figure 7:**
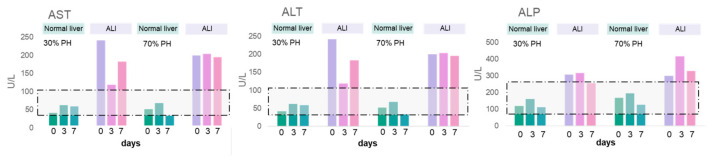
Biochemical analysis of the level of liver damage markers in the blood serum of rats during regeneration with concomitant ALI. The areas marked with a dotted line reflect the range of physiological values for each biochemical parameter under study.

Biochemical blood tests confirmed cell damage during ALI (high values of AST, ALT, and ALP) at all stages of liver regeneration (Figure 7).

We also confirmed a violation of the liver synthetic functions (low values of total protein and globulin). The ALI liver levels of urea and creatinine for 30% PH were within the normal ranges, while in the case of 70% PH, there was a decrease in these parameters. During the regeneration of normal liver, all values of biochemical blood parameters were within the normal ranges. The results are presented in Figure S1.

### 3.5. Real-Time PCR

In the liver with ALI on day 0, the mRNA levels of the genes CAT (16.5-fold), GSS (4.5-fold), CDKN1A (14.4-fold), and UCP2 (3.5-fold) were significantly increased, indicating an induction of a response to oxidative stress [21]. Apparently, PH aggravates oxidative and endoplasmic reticulum (ER) stress during regeneration in the context of ALI and leads to insufficient compensatory processes, which are most pronounced in the 70% PH model.

Compared with the normal liver, in the liver with ALI on the 3rd day of regeneration after 30% PH, the levels of mRNA for the CAT and CDKN1A genes increased by 2.3 and 2.1 times, respectively. On the 7th day of regeneration after 30% PH, the increased level of mRNA for the CAT gene was still two times greater than that on the 7th day of normal liver regeneration.

On the 3rd day after 70% PH with ALI, a 2.4-fold decrease in GSS expression was observed. CAT and UCP2 expression also slightly decreased compared to the 3rd day of normal liver regeneration. By the 7th day, the mRNA level of the CAT gene (1.7-fold) and the CDKN1A gene (2.2-fold) showed an increase compared to the 7th day of normal liver regeneration. Additionally, the levels of mRNA for the UCP2 and CAT genes on the 3rd day after 70% PH were lower by 1.8 times and 2.6 times, respectively, compared to 30% PH in the ALI group.

It is worth noting that in APAP-induced ALI, the depletion of the glutathione pool occurs [22], which leads to disturbances in antioxidant defense and in the regulation of enzymes synthesizing glutathione [23,24,25,26]. This subsequently leads to the inability to compensate for transient ER stress, which is characteristic of liver regeneration [27]. A more significant decrease in the expression levels of antioxidant defense genes in 70% PH compared to the 30% PH model with ALI apparently contributes to the aggravation of pathology.

In the liver with ALI on day 0, the mRNA levels of HGF, EGFR, and IL6R were significantly reduced (3-fold, 9-fold, and 2.7-fold, respectively), while the level of HNF4 was increased 3.6-fold. Increasing the volume of resection contributed to a mild decrease in the expression of most of the analyzed growth factor and receptor genes at the early stages of regeneration. In the case of 70% PH compared to 30% PH, by the 3rd day of regeneration with ALI, a greater decrease in the mRNA levels of the genes EGFR (1.6-fold), HGF (1.5-fold), HNF4 (1.9-fold), PDGFRB (3-fold), and TNFR1 (1.8-fold) was revealed, which also indicate a decrease in the liver regenerative potential [28]. After PH with ALI, the peak of HGF expression was observed on the 7th day but not on the 3rd day, as in the case of normal liver regeneration.

The regeneration of the normal liver was characterized by a sharp increase in the level of TNFR1 mRNA from the 3rd day; however, in the ALI model, an increase was observed only on the 7th day for both 30% PH and 70% PH. Compared with the corresponding time point in normal regeneration, by the 3rd day after 30% PH with ALI, the PDGFRB gene mRNA level had increased by 1.8 times. By the 7th day there was an increase in CCND1 mRNA by 3.9 times after 70% PH with ALI, while the mRNA levels of the HNF4 genes were reduced by 1.8 times, and those of CCND1 by 2.8 times by the 3rd day after 70% PH; by the 7th day, for all genes studied, no statistically significant differences were found.

We also analyzed the expression of three genes regulating the cell cycle: PCNA, CCND1 (G1/S-phase transition), and CDKN1A (G1/S-phase transition blocker). Normal liver regeneration is characterized by an increase in the expression of all three regulators of the cell cycle on the 3rd day after 70% PH [29]. After 30% PH, the expression remains unchanged, which corresponds to two different mechanisms underlying liver regeneration at different volumes of resection [1,15,30]. On 7th day, for both 30% and 70% PH, there was a tendency for decreased CCND1 expression, CDKN1A expression returned to its initial level, and PCNA was slightly elevated. In the ALI group, there was an increase in the expression of CCND1 and CDKN1A, along with a significant decrease in PCNA expression on day 0. On the 3rd day after 30% and 70% PH with ALI, the PCNA and CDKN1A levels remained elevated, but CCND1 did not change after 30% PH and decreased significantly after 70% PH, compared to the normal liver regeneration. On the 7th day, after both 30% and 70% PH, CCND1 expression increased slightly after 30% and 70% PH, while the CDKN1A and PCNA levels practically did not differ from the corresponding point of normal regeneration. Thus, the dynamics of the cell cycle differed significantly during liver regeneration with ALI. According to the literature data, in the case of APAP-induced ALI, cells enter proliferation unevenly [31] and predominantly stop the cell cycle at the checkpoint stage [32], and on the 3rd day there is a blockage of the G1/S transition [33,34,35,36].

**Figure 8:**
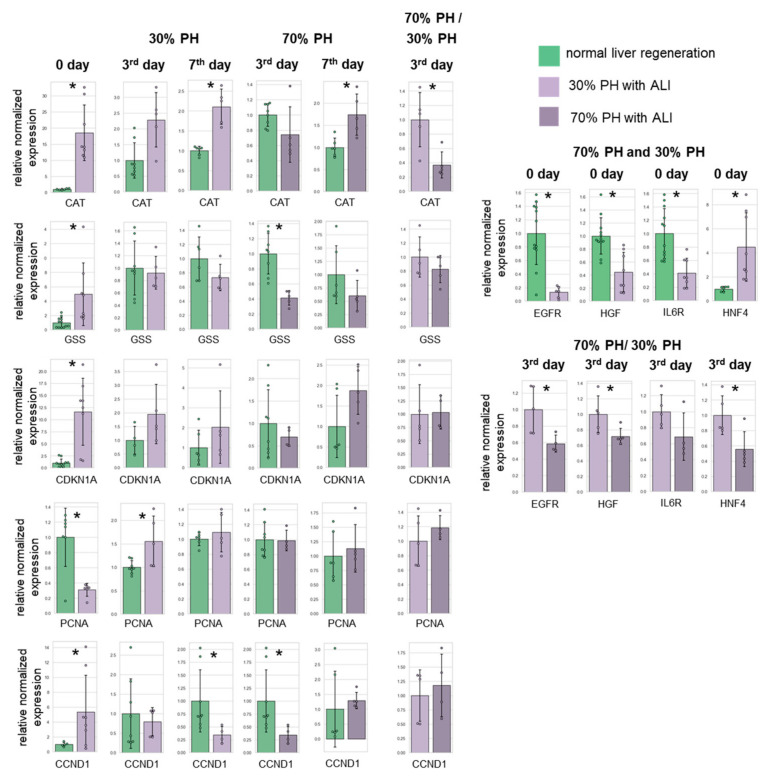
Analysis of changes in gene expression during normal regeneration and during regeneration with ALI. *—statistical differences for time points of normal regeneration from the corresponding time points of regeneration with ALI; *p*-value ≤ 0.05.

The results of the analysis of the expression of genes associated with liver regeneration and liver regeneration with ALI are presented in Figure 8 and Figure S2.

Thus, the data from this study attest to a decrease in the regenerative potential of the liver in the presence of concomitant ALI, which corresponds to the results obtained using multiphoton microscopy, SHG, and FLIM.

## 4. Discussion

To reduce the risks of developing postoperative liver failure, it is necessary to obtain new fundamental data and new criteria for determining the decrease in the regenerative potential of the liver. ALI, characterized by a rapid loss of functional hepatocytes, is a suitable model for this task. If not treated promptly, it can easily lead to liver failure and a lethal outcome within days or weeks, which is currently lacking effective treatment [21]. Our newly identified imaging criteria for the assessment of the metabolic state of hepatocytes are promising for the rapid assessment of the regenerative potential of liver tissue before surgery.

Earlier, we demonstrated that in a healthy liver, OXPHOS is the essential metabolic pathway in hepatocytes. During the process of physiological (normal) regeneration, on the 3rd day following PH, there is a significant surge in the contribution of the bound forms of NADH and NADPH. This indicates an even greater rise in the intensity of OXPHOS and synthetic processes, due to the heightened energy requirements of the proliferating hepatocytes [15,16]. However, most patients undergoing major resections have some degree of concomitant acute or chronic liver disease, which significantly reduces the liver’s ability to recover after surgery. In this regard, it is necessary to study the metabolic mechanisms that underlie impaired liver regeneration in pathology [1,28]. The key mechanism of pathogenesis of almost any liver disease is mitochondrial dysfunction, so interest in its study within the context of impaired liver recovery has been increasing in recent years. Extensive research into the source of the oxygen free radicals occurring in ALI has now established that mitochondrial dysfunction is pivotal for hepatocyte necrosis [37]. It is clear that changes in the intensity of the main pathways for obtaining energy within cells are sensitive, dynamically changing markers for the condition of the liver at the organ level [21,38]. Emerging evidence indicates that mitochondria play a crucial role in liver regeneration following acute liver injuries. After PH, mitochondria facilitate hepatocyte proliferation by generating ATP through OXPHOS to fulfill the bioenergetic needs of the hepatocytes [30,39]. The onset of mitochondrial dysfunction leads to reactive oxygen species (ROS), depletion of glutathione (GSH), protein alkylation, and alterations in respiratory complexes, ultimately resulting in cell death. Depending on their characteristics and severity, these mitochondrial changes can cause lipid accumulation, apoptosis, and/or necrosis, resulting in hepatic cytolysis and inflammation [40]. The pathological depletion of glutathione occurs as a response to ROS accumulation in damaged cells, which is a common pathological feature in liver diseases [41]. Using FLIM, we have confirmed the pathological metabolic alterations occurring in hepatocytes, including a reduction in OXPHOS intensity, a decrease in the intensity of the glutathione cycle, and a decline in biosynthetic processes by the 3rd day of liver regeneration. Furthermore, the development of oxidative stress is supported by molecular analysis results, which revealed an increase in the expression of genes related to the activation of antioxidant defense following ALI induction.

The identification of mitochondrial regulators and a deeper understanding of their regulation on mitochondria may pave the way for the future development of therapeutic strategies in liver injury. The regulation of mitochondrial homeostasis is now increasingly being considered as a therapy for a range of diseases, including steatosis, steatohepatitis, and fibrosis, as well as a therapy to promote liver repair following injury. The optical criteria obtained in this work offer opportunities as diagnostic tools for dynamic, rapid monitoring of the effectiveness of such therapy [37].

## 5. Conclusions

In this work, using the methods of multiphoton microscopy with SHG and FLIM, we determined optical and metabolic changes characteristic of a critical decrease in the regenerative potential of the liver in a model of acute liver injury. These include the following: (1) the appearance of zones with reduced intensity of NAD(P)H fluorescence, associated with damaged hepatocytes and foci of fibrosis; (2) extensive areas characterized by the second harmonic generation signal of collagen; (3) the absence of a sharp increase in the values of the contributions of the fluorescence lifetimes of the bound forms of NADH (a2) and NADPH (a3) on the 3rd day of regeneration. The results obtained have both fundamental and applied significance and should be useful for the rapid intraoperative assessment of the condition of the liver during resection. In addition, tracking such data should serve to monitor the effectiveness of therapy of liver pathology and its role in stimulating liver recovery.

## Data Availability

Data available on request from the corresponding author.

## Supplementary Materials

The following supporting information can be downloaded at: https://www.mdpi.com/article/10.3390/cells13232015/s1. Figure S1: Biochemical parameters in the blood serum of rats during regeneration with concomitant ALI. The areas marked with a dotted line reflects the range of physiological values for each biochemical parameter under study. Figure S2: Analysis of changes in gene expression during normal regeneration and during regeneration with ALI. *—statistical differences for time points of normal regeneration from the corresponding time points of regeneration with ALI; *p*-value ≤ 0.05. Table S1: Analysis of the recovery of liver weight. Table S2: The primer sequences for RT-PCR.
