# Supplementary material for: Novel Optical Criteria and Mechanisms of Critical Decline in Liver Regenerative Potential
Source: Cells. 2024 Dec 5;13(23):2015. doi: 10.3390/cells13232015 (PMC11639982; doi:10.3390/cells13232015)
Supplement: Supplementary file 1 [file cells-13-02015-s001.zip › cells-3263858-supplementary.pdf]

Supplementary Materials

Novel Optical Criteria and Mechanisms of Critical Decline in the Liver Regenerative Potential

Svetlana Rodimova [1], Vera Kozlova [1,2], Dmitry Kuzmin [1,2], Nikolai Bobrov [3], Artem Mozherov [4], Vadim Elagin [1], Ilya Shchechkin [1,2], Dmitry Kozlov [1,4], Alena Gavrina [1], Vladimir Zagainov [5], Elena Zagaynova [1,6] and Daria Kuznetsova [1,4,*]

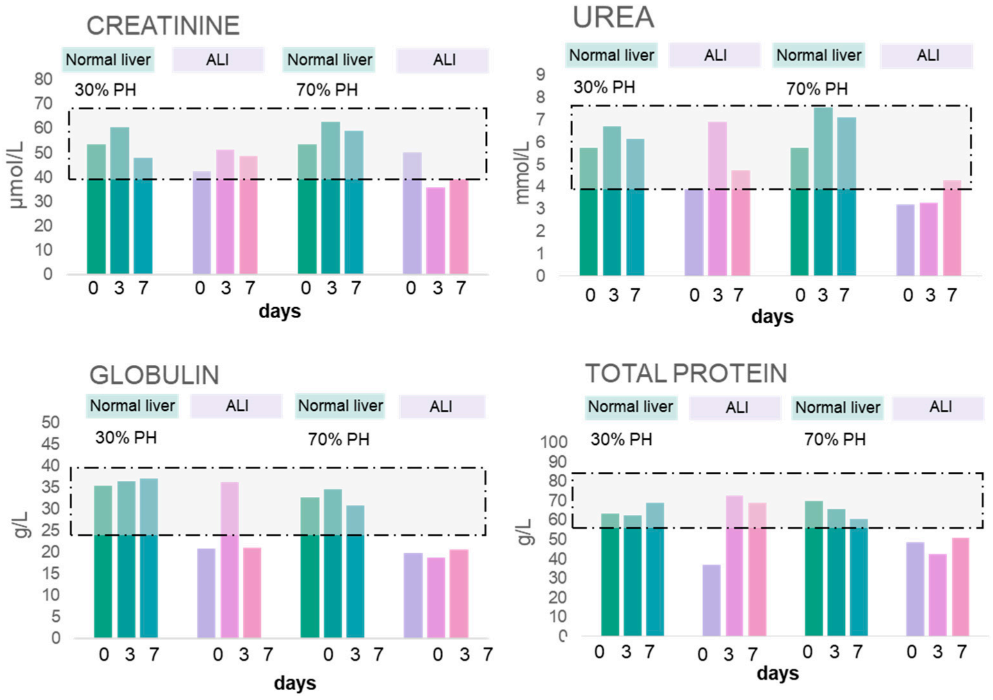

Figure S1. Biochemical parameters in the blood serum of rats during regeneration with concomitant ALI. The areas marked with a dotted line reflects the range of physiological values for each biochemical parameter under study.

Table S1. Analysis of the recovery of liver weight.

	Normal regeneration				ALI			
	30% PH		70% PH		30% PH		70% PH	
	3rd day	7th day	3rd day	7th day	3rd day	7th day	3rd day	7th day
absolute weight(%)	83,4	88,4	80,9	92,9	86,8	80,2	74,0	87,6
relative weight(%)	-	-	-	-	104,1	90,7	91,5	94,2

Table S2. The primer sequences for RT-PCR.

Primer Target	Primer Sequence
CAT	F: GCTCCGCAATCCTACACCAT<br>R: GGACATCGGGTTTCTGAGGG
CCND1	F: CGTACCCTGACACCAATCTCC<br>R: TCTGCTCCTCGCAGACCTCTA
CDKN1A	F: AAAACGGAGGCAGACCAG<br>R: ACTTCAGGGCTTTCTCTTGC
CTGF	F: GCTGGAGAAGCAGAGTCGTC<br>R: ATGCACTTTTTGCCCTTCTTAATG
CYP2E1	F: AGGCTGTCAAGGAGGTGCTACT<br>R: AAAACCTCCGCACGTCCTTCCA
CYP7A1	F: CTGCCGGTACTAGACAGCATC<br>R: CCGTCCTCAAGATGGAGAGTG
EGFR	F: ATGTCAACAACCAGAAGGGCCAA<br>R: GTGACGCCTTCGCATGAAGA
GSL	F: AAGCATAGCATTTAGATTGCG<br>R: CTGTCCAAGTTCATCACCACTT
GSS	F: CCGAAGGCTGTTTATGGAGGA<br>R: GATCTCTCTAGCAGCAGGCG
HGF	F: TTGCCCTATTTCCCGTTGTGA<br>R: ACCATCCACCCTACTGTTGT
HNF4	F: CGACTCTCTAAAACCCTCGCC<br>R: CAGATGGGGATGTGTCATTGCCC
HPRT	F: CTCATGGACTGATTATGGACAGGAC<br>R: GCAGGTCAGCAAAGAACTTATAGCC
il6R	F: AAGCAGGTCCAGCCACAATGTAG<br>R: CCAACTGACTTTGAGCCAACGAG
PDGFRB	F: TAGCACACATCAGGAGCCATC<br>R: CGAACAGCAACAATTGGCCTCT
TNFR1	F: CCAAGTGCCACAAAGGAACC<br>R: CCGACATGTCTTGCAACTGAG
UCP2	F: TCTCCCAATGTTGCCCGAAA<br>R: CAAGGGAGGTCGTCTGTCAT

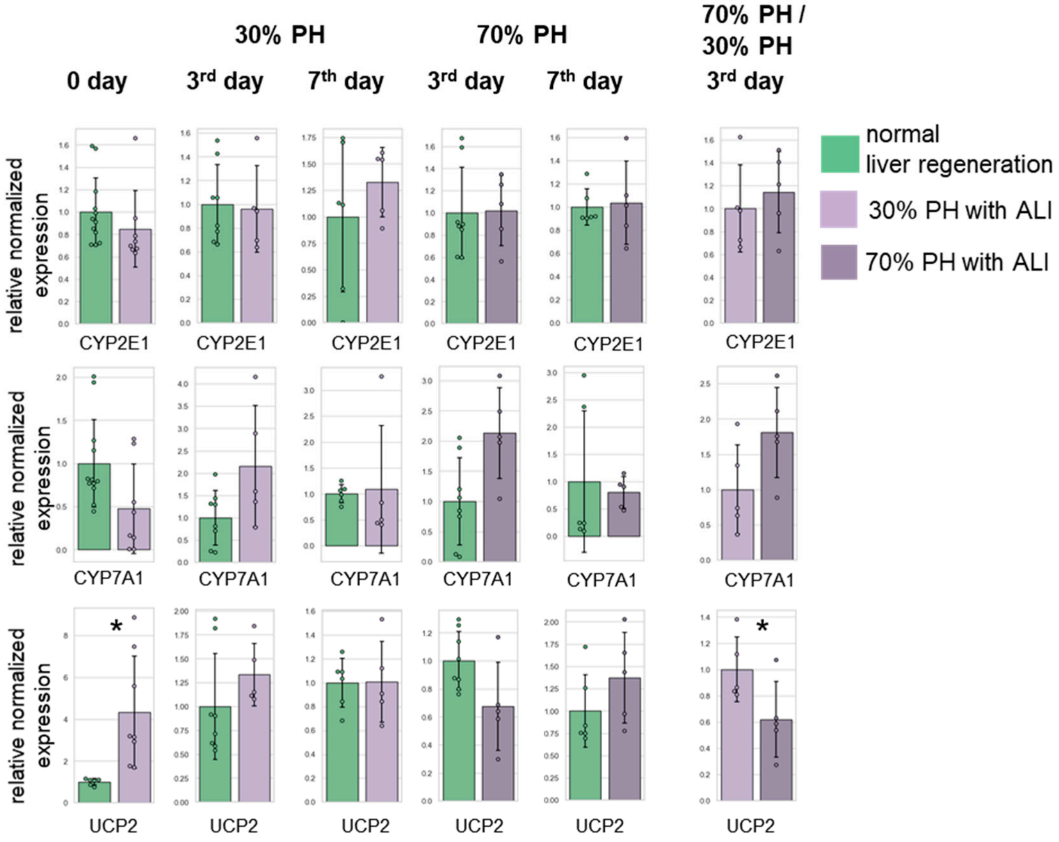

Figure S2. Analysis of changes in gene expression during normal regeneration and during regeneration with ALI. *—statistical differences for time points of normal regeneration from the corresponding time points of regeneration with ALI; p-value ≤ 0.05.
